# Kiss1 gene expression, sperm indices and testicular histopathology following the administration of *Hibiscus sabdariffa* in rats

**DOI:** 10.5935/1518-0557.20220029

**Published:** 2023

**Authors:** Izuchukwu Azuka Okafor, Uchenna Somtochukwu Okafor, Johnson Okwudili Nweke, Kingsley Chinemerem Ibeabuchi

**Affiliations:** 1 Department of Anatomy, Faculty of Basic Medical Sciences, College of Health Sciences, Nnamdi Azikiwe University, Nnewi Campus, PMB 5001, Nnewi, Nigeria; 2 Department of Obstetrics and Gynaecology, College of Medicine, University of Ibadan, Ibadan, Nigeria; 3 Pan African University of Life and Earth Science Institute (Including Health and Agriculture), PAULESI, University of Ibadan, Ibadan, Nigeria; 4 Hematology Department, Babcock University Teaching Hospital, Ilisan-Remo, Ogun State, Nigeria; 5 Diagnostic Laboratory Unit, Medical Centre, Michael Okpara University of Agriculture, Umudike, Abia State, Nigeria; 6 College of Nursing Sciences, Our Lady of Lourdes Hospital Complex, Ihiala, Anambra State, Nigeria

**Keywords:** Kiss1 gene, relative gene expression, Hibiscus sabdariffa, sperm parameters, antioxidants, reproductive hormones

## Abstract

**Objective:**

This study investigated the expression of Kiss1 gene on the testis and the blood of Wistar rats, following the administration of methanolic extract of *Hibiscus Sabdariffa* (MEHS).

**Methods:**

Fifteen (15) rats with an average weight of 204g were randomly divided into three (3) groups (A-C). Group A was given no treatment and served as the normal control group. Groups B and C were orally administered 200mg/kg and 400mg/kg of MEHS, respectively. The extract was administered once a day for 21 days.

**Results:**

There was a significant increase in the relative testicular weight in group B and C compared to the control group (*p*=0.035). There was no significant difference in the sperm parameters, reproductive hormones, and antioxidant levels in all the treatment groups when compared to the control group (*p*>0.05). There is a significantly lower expression intensity of the Kiss1 gene in the blood in groups B (*p*=0.000) and C (*p*=0.017), compared to the control group. There is no difference in the relative intensity of Kiss1 gene expression in the testis of all the experimental groups (*p*=0.173).

**Conclusions:**

MEHS caused no histopathological changes on the testis at both doses. MEHS shows the potential of downregulating the expression of the Kiss1 gene in the blood. However, this effect lacks a regulatory mechanism on the reproductive hormones, sperm parameters, testicular morphology, and antioxidative levels.

## INTRODUCTION

*Hibiscus sabdariffa (HS)* is a medicinal plant from Egypt (Ali *et al*., 2005). It is well known for its fleshy red calyces used for the production of soft drinks and tonic without alcohol, like juice, jam, jelly, syrup and also dried and brewed into tea and spices (Ismail *et al*., 2008). These are rich in carotene, riboflavin, anthocyanins, ascorbic acid, niacin, calcium, iron and vitamin C (Cisse *et al*., 2009). Phytochemical analysis of HS shows a rich content of different nutrients, with a significant high amount of protein, dietary fiber, vitamins, and minerals, as well as bioactive compounds like anthocyanin and flavonoids (Ismail *et al*., 2008; Okereke *et al*., 2015). The young leaves and tender stems of Roselle are consumed raw, as green vegetable. Roselle seeds are a good source of protein, fat, total sugars, and are widely used in the diet of many African countries (Mahadevan *et al*., 2009). Even though Roselle has many and varied uses both in food and in traditional medicine, all parts of Roselle including seeds, leaves, fruits and roots are used as food in different parts of the world (Qi *et al*., 2005). All parts of HS have been used for medicinal purposes in traditional healing to mitigate ailments such as cough, diabetes, fever, and heart diseases (Qi *et al.,* 2005). It boasts antioxidant, antibacterial (Mak *et al*., 2013; Mokhtari *et al*., 2018), anti-hypertensive, hypolipidemic (Hopkins *et al*., 2013), and diuretic effects (Alarcón-Alonso *et al*., 2012).

Kiss1 is a cancer suppressor gene, well-known to inhibit the metastasis of malignant melanomas and breast cancer, often by inhibiting chemotaxis and invasion (Kauffman, 2009). In addition to its role in preventing cancer metastasis, the Kiss1 gene encodes a family of neuropeptides called Kisspeptins (kisspeptin 13 and 14), involved in the neuroendocrine initiation of puberty (Tena-Sempere, 2010; Rhie, 2013). Kisspeptin actively stimulates the release of Gonadotropin-releasing hormone by binding to the G-protein coupled receptor GPR54 (also called Kiss1r), which is expressed by GnRH neurons (Ruohonen *et al*., 2020). The secretion of gonadotropin-releasing hormone triggers LH and FSH release, leading to sexual maturation (Ruohonen *et al*., 2020). Kisspeptin neurons are located in the arcuate (ARC) and anteroventral periventricular (AVPV) nuclei of the hypothalamus, where they produce kisspeptin, which binds to the Kiss1r on GnRH neurons, stimulating the secretion of GnRH (Hu *et al*., 2018). Studies have shown the Kiss1 gene to be expressed locally in peripheral tissues like the ovary (Han *et al*., 2020), testis (Aloqaily *et al*., 2020), liver (Dudek *et al*., 2016), fat, pancreas, and liver (Hsu *et al*., 2014). Although the specific reproductive actions of the Kiss1 gene is still unclear, studies have demonstrated a possible modulatory effect of the locally derived Kiss1 gene on peripheral reproductive tissues. The Testicular Kiss1 gene is said to have direct stimulatory effect on the secretion of testosterone by Leydig cells (Aloqaily *et al*., 2020). It alters the intracellular levels of calcium ions in sperm cells (García-Galiano *et al*., 2012). While the Kiss1 gene is mainly regulated by endogenous sex steroids (Fan *et al*., 2015), some exogenous phytochemicals may alter the level of Kiss1 gene expression, induce hormonal changes, and affect puberty (Okafor *et al*., 2014; 2020a). Here, we studied the changes in the Kiss1 gene expression in the blood and the testis of adult male Wistar rats following the administration of methanolic extract of *MEHS.*

## MATERIALS AND METHODS

### Study setting

This experimental study was carried out in the research laboratory of the Department of Anatomy, School of Basic Medical Sciences, College of Health Science, Nnamdi Azikiwe University, and lasted for about three months.

### Plant collection, identification, and extraction

The dried aerial parts of HS were procured from the local market at Nnewi, Anambra state, Nigeria. The botanical identification and authentication were carried out in the Department of Pharmacognosy and Traditional Medicine, College of Pharmacy, Nnamdi Azikiwe University, with identification number PCG/1474/A/031. The plant calyces were shade-dried and ground. 1000g of powdered plant sample was used for methanolic extraction, as described in a previous study (Okafor & Gbotolorun, 2018). The filtrate (extract) was then stored in the refrigerator at 4^o^C. The extract was brought to a solution at varying doses per ml on each administration day and given according to body weight and group treatment doses.

### Animal procurement, Care and Handling

Fifteen (15) male Wistar rats were procured from the animal housing facility of the College of Health Sciences, Nnamdi Azikiwe University and acclimatized for two (2) weeks (to rule out any intercurrent infection) under standard housing condition (ventilated room with o-12/12-hour light/dark cycle at 24±2^o^C). The rats were fed ad libitum with water and standard rat chow throughout the experimental period. According to the federation of European Laboratory Animal Science Associations (FELASA) guidelines, the animals’ health statuses were monitored throughout the experiment.

### Experimental design

Fifteen (15) 11-week-old rats with an average weight of 204 g were randomly divided into three (3) groups (n=5), A-C. Group A was given no treatment and served as the normal control group. Groups B and C received oral administration of 200mg/kg and 400mg/kg of MEHS, respectively. The doses of MEHS used for this study were determined based on a previous study (Okafor *et al*., 2020b). The extract was administered once a day for 21 days.

### Animal Slaughter and Sample Collection

The animals were fasted overnight on the last day of MEHS administration and anaesthetized using chloroform 2 ml of blood was collected from the animals by ocular puncture using capillary tubes into two different sample tubes. One was collected into a plain tube for hormonal assay, and the other into an RNA protector-containing plain tubes for Kiss1 gene analysis. The animals were slaughtered after blood collection, and the testicular tissues were harvested, weighed, and divided into three parts. One part was fixed in a 10% formal saline for histological processing and analysis. The second part was homogenized and used for oxidative status analysis. The last part was stored in an RNA protector-containing plain tube before RNA isolation. The epididymis was also harvested for Epididymal sperm extraction and analysis.

### Epididymal Sperm Analysis

Following the epididymis’ removal, the spermatozoa were squeezed into a petri dish containing 5 ml of saline at 37^o^C. 0.5 ml of the epididymal fluid was then added to 1ml of the semen diluting fluid and mixed thoroughly. One drop of the diluted epididymal fluid was added to the hemocytometer for 10 minutes. Sperm count, sperm motility, and sperm morphology were determined by the method described by Srinivasulu & Changamma (2017).

### Hormonal Assay

The blood was allowed to clot and centrifuged at 5,000 rpm for 10 minutes within one hour after collection. The serum was extracted and used for the hormonal assay. AccuBind enzyme-linked immunoabsorbent assay (ELISA) microwells for Follicle-stimulating hormone (FSH), Luteinizing hormone (LH), and Testosterone (TT) were purchased from Calibiotech Inc. (catalogue number: E5380s), Bioassay technology laboratory China (catalogue number: EO182Ra), and Bioassay technology laboratory (catalogue number EO179Ra) respectively. All the analyses were carried out following the accompanying ELISA kit protocol for each parameter.

### Testicular Oxidant Status

Superoxide Dismutase (SOD), Glutathione (GSH), and Catalase (CAT) levels were quantified in the testicular tissues to determine the oxidant status using the tissue homogenate derived from one part of the testis. The protocol used for this was described in our previous study (Carvajal-Zarrabal *et al*., 2009).

### Kiss1 RNA Extraction

According to Zymo Research (ZR) specifications, Total RNA was extracted using the ZR whole-blood RNA MiniPrep. A 600 µl volume of red blood cell lysis buffers were added to 200 µl volume of ribonucleic acid-guard (RNAguard) stored whole blood sample in an RNase-free tube and mixed by inverting. The mixture was incubated for 5 minutes at 25°C and centrifuged at ≥ 12,000 × g for 1 minute. The supernatant was removed. A 600 µl volume of blood RNA buffer was added to the cell pellet and mixed properly. The resultant mixture was transferred into the Zymo-Spin IIIC column in a collection tube and centrifuged at ≥ 12,000 × g for 2 minutes. The column was transferred into a new collection tube. A 400 µl volume of RNA pre-wash buffer was added to the column and centrifuged at ≥ 12,000 × g for 30 seconds. The column was transferred to an RNase-free tube. 100 µl RNA recovery buffer was added to the Zymo spin IIIC column and centrifuged at ≥ 12,000 × g for 30 seconds. A 100 µl volume ethanol (100%) was added to the RNase free tube flow-through and mixed by pipetting. The mixture was transferred into the Zymo spin IC column in a collection tube and centrifuged at ≥ 12,000 × g for 30 seconds. A 400 µl volume of the RNA prep buffer was added to the column and centrifuged at ≥ 12,000 × g for 1 minute; the flow-through was discarded. An 800 µl volume of the RNA wash buffer was added to the column and centrifuged at ≥ 12,000 × g for 1 minute; the flow-through was discarded. The wash step was repeated with 400 µl volume of RNA wash buffer. The Zymo-spin IC column was centrifuged in an empty collection tube at ≥ 12,000 × g for 2 minutes. It was then transferred into an RNase-free tube. Total RNA was eluted by added 80 µl volume of DNase/RNase free water directly to the column matrix and centrifuged at 10,000 × g for 30 seconds. A 70 µl volume of the Total RNA extracted was transferred into an RNA stable tube supplied by Biomatrica (catalogue number 93221-001) to store Total RNA at room temperature, while 10 µl was used for quality control check on the total RNA extracted. The above-described procedure was also followed for RNA extraction from the testicular tissue homogenate.

### RNA Detection

One gram of agarose powder was weighed and poured into 100 ml of Tris EDTA buffer in a Pyrex conical flask. It was heated using a microwave at 100°C for 5 minutes. It was allowed to cool to 56°C, and 6 µl volume of ethidium bromide was added to 100 ml of the gel mixture. The gel was poured into the electrophoresis chamber and allowed to solidify. A 3 µl volume of loading dye was added to 7 µl volume of the Total RNA from each sample; the molecular marker was loaded in the first lane, followed by the samples. Electrophoresis was performed at 90 volts for 30 minutes. The gel was removed and viewed on the UV transilluminator; a picture of the gel was taken.

### Reverse transcriptase-polymerase chain reaction (RT-PCR)

The extracted total RNA was retro-transcribed and amplified using One Taq one-Step RT-PCR kit (catalogue number NEB E5315S) by New England BioLabs incorporation according to the manufacturer’s specification. We used selected primers to target the specific genes using the MJ research Peltier thermal cycler polymerase chain reaction machine. The PCR was performed in a 50 µl volume reaction mixture containing 25 µl volume of one Taq one-step reaction master mix (2x), 2 µl volume of One Taq one-step enzyme mix (2x), 2 µl volume of each gene-specific forward primer (10 µM), 2 µl volume of each gene-specific reverse primer (10 µM), 9 µl volume of nuclease-free water and 10 µl volume of the RNA template was added. Negative control samples for the RT-PCR consisted of a mixture to which all reagents were added, except the RNA. The PCR was started as follows: Reverse transcriptase at 48°C for 30 seconds, initial denaturation at 94°C for 1 minute, denaturation at 94°C for 15 seconds, annealing at Tm° C-5 (the lowest melting temperature of each set of Kiss1 gene) for 30 seconds, extension at 68°C for 1 minute, denaturation step for 39 cycles, final extension at 68°C for 5 minutes and final holding at 4°C, indefinitely. The Kiss1 gene nucleotide sequence (5’-3’) for the primers are as follows: forward primer - CTACGACTCCTTGTTGCTTTG, and reverse primer - TGATCTTCACTGTAGTTGGTGG.

### Electrophoresis

5 µl of the amplified PCR products and DNA ladder were analyzed on 1% agarose gel containing ethidium bromide in 1X Tris EDTA buffer. One percent agarose gel was prepared by dissolving 1.0 g of LE agarose powder in 100 ml volume of Tris Borate EDTA Buffer. The mixture was then heated in a microwave at 100°C for 5 minutes and allowed to cool to 56°C, and 6 µl volume of ethidium bromide was added to it. The agarose gel was poured into the electrophoresis chambers with a gel comb and allowed to solidify. Electrophoresis was performed at 90 volts for 30 minutes with the EDVOTEK tetra source electrophoresis machine, Bethesda, USA, and the Kiss1 gene expression was visualized using the Wealtec Dolphin-Doc UV transilluminator and photographed.

### Kiss1 Gene Relative Intensity of Expression

We used the ImageJ 1.53a software to calculate the absolute intensity of expression from the generated gel images across all the experimental groups in both the blood and testis. ImageJ generates the absolute intensity (derived by the mean value multiplied by each band’s pixel value or percent). The absolute intensity is an integrated measure of the intensity and size of the band. The relative intensity was calculated by dividing the absolute intensity of each sample band by the absolute intensity of the standard.

### Tissue processing

The testicular tissue samples were trimmed down to about 3mm x 3mm thick for an easy study of sections under the light microscope and fixed in 10% formalin. After fixation, the fixed tissues’ dehydration was done in ascending grades of alcohol, 50%, 70%, 95% and 100%, and cleared in xylene. Staining was done with hematoxylin and eosin (H&E), and mounted using DPX, after which the sections were viewed under the light microscope. Photomicrographs of these sections were obtained using the Leica DM 750 digital light microscope photomicrography computer software.

### Statistical analysis

The data were analyzed using IBM statistical package for social sciences (SPSS) for Windows, version 23 (IBM Corporation, Armonk, New York, USA). One-way analysis of variance (ANOVA), post hoc LSD, student’s t-test and Pearson’s correlation analysis were used to test for significance in changes seen in the variables across groups. Tables and figures were used to present the data, and values were considered significant at *p*<0.05.

## RESULTS

### The effect of MEHS on rat body weight

Table 1 shows a significant (*p*<0.05) decrease in the body weight of animals in group A, while animals in groups B and C showed no significant (*p*>0.05) changes in their body weights when the pre and post-administration body weight were compared.

**Table 1:** The effect of MEHS on the bodyweight of Wistar rats.

Groups		Mean±SD (g)	*p*-value
A (control)	Pre-administration Post-administration	252.00±26.83 222.00±17.89	0.003^*^
B (MEHS 200)	Pre-administration Post-administration	170.00±32.60 162.00±40.25	0.347
C (MEHS 400)	Pre-administration Post-administration	190.00±2.36 200.00±18.71	0.210

Data were analyzed using the Students' dependent t-test. The values were expressed as mean ± standard deviation, and values were considered significant at

* *p*<0.05.

### The effect of MEHS on rat relative testicular weight

The right testis’ relative testicular weight showed a significant (*p*<0.05) increase when the MEHS-treated groups were compared to the control group. In contrast, the left testis showed no significant difference when the MEHS-treated groups were compared to the control (*p*>0.05) (Table 2).

**Table 2:** The effect of MEHS on the relative testicular weight of the Wistar rat.

	Groups	MEAN ±SD	*p*-VALUE
Right testis	A (CONTROL) B (MEHS 200) C (MEHS 400)	0.006±0.001 0.008±0.001 0.008±0.001	0.035^*^
Left testis	A (CONTROL) B (MEHS 200) C (MEHS 400)	0.007±0.003 0.008±0.001 0.008±0.001	0.729

Data were analyzed using one-way ANOVA, followed by multiple comparisons using LSD and data were considered significant at

* *p*<0.05.

### The effect of MEHS on the Epididymal sperm parameters

There were no significant changes in sperm count, sperm motility, and sperm morphology when the MEHS-treated groups were compared to the control group (*p*<0.05) (Table 3).

**Table 3:** The effect of MEHS on the Epididymal sperm parameters of Wistar rat.

	Groups	Mean ±SD	*p*-value
Sperm motility (%)	A (CONTROL) B (MEHS 200) C (MEHS 400)	71.00±15.17 85.00±0.00 85.00±0.00	0.057
Sperm count (10×6)	A (CONTROL) B (MEHS 200) C (MEHS 400)	34.84±16.70 42.58±13.87 39.70±2.18	0.650
Sperm Morphology (%)	A (CONTROL) B (MEHS 200) C (MEHS 400)	89.00±2.24 88.75±2.50 89.40±2.61	0.922

Data were analyzed using One-way ANOVA and values were considered significant at *p*<0.05.

### The effect of MEHS on male reproductive hormones

Table 4 showed no significant changes in the LH, FSH and TT levels when the MESH-treated groups were compared to the control group (*p*>0.05).

**Table 4:** The effect of MEHS on LH, FSH and TT in Wistar rats.

	Groups	MEAN±SD	*p*-value
LH (IU/L)	A (CONTROL) B (MEHS 200) C (MEHS 400)	6.18±1.87 7.20±0.96 7.83±3.51	0.646
FSH (mIU/ml)	A (CONTROL) B (MEHS 200) C (MEHS 400)	5.50±2.65 8.77±3.87 5.07±0.29	0. 245
TT (ng/ml)	A (CONTROL) B (MEHS 200) C (MEHS 400)	1.07±1.70 1.30±1.71 0.87±1.31	0.492

Data were analyzed using one-way ANOVA. Values were considered significant at *p*<0.05. LH - luteinizing hormone; FSH - Follicle-stimulating hormone; TT - Testosterone.

### The effect of MEHS on the antioxidant levels in the testis

There were no significant differences in SOD, GSH and CAT levels in the testis when MEHS-treated groups were compared to the control group (Table 5).

**Table 5:** The effect of MEHS on testicular oxidative stress parameters.

Antioxidants	Groups	MEAN±SD	*p*-value
SOD	A (CONTROL) B (MEHS 200) C (MEHS 400)	14.80±0.57 11.56±1.91 12.53±1.81	0.246
GSH	A (CONTROL) B (MEHS 200) C (MEHS 400)	17.82±7.70 25.09±9.39 18.67±5.06	0.627
CAT	A (CONTROL) B (MEHS 200) C (MEHS 400)	71.07±11.67 59.82±5.91 53.37±13.95	0.389

Data were analyzed using one-way ANOVA, followed by multiple comparisons using LSD. SOD - Superoxide dismutase; GSH - Glutathione; CAT - Catalase. Values were considered significant at *p*<0.05.

* *p*<0.05 means a significant difference between groups.

### The relationship between testicular and blood Kiss1 gene expression, reproductive hormones and sperm parameters

Table 6 shows no significant correlation between the relative intensity of Kiss1 expression in the testis, the relative intensity of Kiss1 expression in the blood, the reproductive hormones, and sperm parameters (*p*>0.05).

**Table 6:** Correlation between the relative intensity of Kiss1 gene expression, hormonal levels and sperm indices.

		MOTILITY (%)	COUNT (x10^6^)	MORPHOLOGY (%)	LH (mIU/ml)	FSH (mIU/ml)	TT (ng/ml)
RIB	Sig. (2-tailed)	.094	.515	.785	.172	.880	.266
	Pearson Correlation	-.180	-.207	.331	-.133	.300	-.732
RIT	Sig. (2-tailed)	.699	.655	.469	.831	.624	.061
	Pearson Correlation	.458	.391	.643	1	-.213	.760^*^

RIB: Relative expression intensity in the blood; RIT: Relative expression intensity in the Testis; LH: luteinizing hormone; FSH: Follicle-stimulating hormone; TT: Testosterone;

* Correlation is significant at the 0.05 level (2-tailed).

### Testicular Histopathology

**Figure 1:**
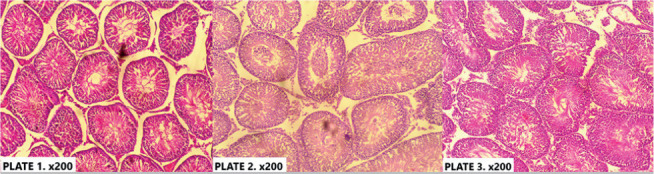
The histological section of rat testis administered distilled water (plate 1), 200 mg/kg of MEHS (plate 2) and 400 mg/kg of MEHS (plate 3) for 21 days. H&E, x200.

Figure 1 (Plate 1-3) shows the photomicrograph of the testis of all experimental animals. Plate I represents the control group (group A), showing the histological section of the testis of rat administered only distilled water for 21 days. The micrograph shows normal testis histology. Plate 2 (group B) represents the histological section of rat testis administered 200 mg/kg of MEHS for 21 days. It showed a normal testicular histoarchitecture with visible spermatogenic cell series and luminal spermatozoa in the tubules. Plate 3 (group C) represents the histological section of rat testis administered 400 mg/kg of MEHS for 21 days. The section shows normal tubules lined by spermatogenic series with numerous luminal spermatozoa. Staining for all sections was done using H&E, and photomicrography was taken at x200.

### Kiss1 gene expression on the testis and blood of male Wistar rats

**Figure 2:**
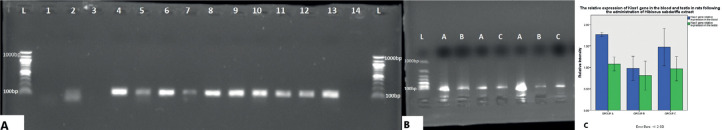
(A-C). RT-PCR gel images showing the detection of Kiss1 gene in the blood (A), the testis (B) in male Wistar rats and their relative expression (C). Fig. 2A - samples 2, 4, 5, 6, 7, 8, 9, 10, 11, 12 and 13 positive bands for the expressed Kiss1 genes at 100bp. 4 represents group A, 2 represents group B, 1 and 3 represents group C. Fig. 2B - samples A, B and C are positive bands for the expressed Kiss1 genes at 100bp. A represents group A, B represents group B and C represents group C. L is a 100bp-1000bp DNA ladder (molecular marker).

Figure 2 (A-C) indicates RT-PCR band images showing the Kiss1 gene in both the blood and the testis of Wistar rats in all the experimental groups and the relative Kiss1 gene expression. Figures 2A and 2B shows an RT-PCR image for Kiss1 gene expression on the blood and testis of an adult male Wistar rat analyzed on a 1.0% agarose gel electrophoresis stained with ethidium bromide, respectively. Kiss1 gene was detected in both the blood and testis across all the groups but at different intensities. Plate C shows the relative intensity of Kiss1 gene expression in the blood and the testis in each experimental group. There is a significantly lower intensity of the Kiss1 gene expression in the blood in groups B (*p*=0.000) and C (*p*=0.017) compared to the control group. There is no difference in the relative intensity of Kiss1 gene expression in the testis of all the experimental groups (*p*=0.173). On comparing the detection levels of the Kiss1 gene at the different doses of MEHS in the two tissues, a significantly higher intensity of Kiss1 gene expression was observed in the blood in group C compared to the group C testis (A=CI: 0.49-0.82, *p*=0.000; B = CI: -0.65-0.96, *p*=0.395; C= CI:-0.19-0.92, *p*=0.045) (Figure 2C).

## DISCUSSION

This study showed no significant difference in the body weight of animals treated with MEHS when the pre and post-administration body weights were compared, although the bodyweights of the control animals were increased significantly (*p*=0.003) (Table 1). This finding corroborates previous studies which reported HS to be effective in causing body weight loss (Ojulari *et al*., 2019; Sellers *et al*., 2007).

There was a significant increase in the relative testicular weight of the right testis in groups B and C (*p*=0.035) compared to the control. While an excessive increase in organ weight might indicate organ toxicity (Katagiri *et al*., 2015), histopathological findings showed no sign of tissue toxicity or damage across all the groups when compared to the control. Similarly, the oxidative stress status of the testis was unaffected, as seen in the unchanged levels of SOD, CAT and GSH, which may indicate the relative safety associated with HS consumption.

Based on our findings, treatment with MEHS did not cause any significant change in the expression of the testicular Kiss1 gene. As seen in Figure 2, the relative intensity of Kiss1 gene expression in the testis was not significantly different in groups B and C compared to the control (*p*=0.173). However, there was a down-regulation in the expression of the Kiss1 gene in the blood, as reflected by the significant decrease in the relative intensity of expression in groups B (*p*=0.000) and C (*p*=0.017) when compared to the control group (Figure 2).

It is interesting to note that the changes in the expression of the Kiss1 gene in the blood did not cause any significant change in the hormonal levels (Table 4) and sperm parameters (Table 3), as both hormonal and epididymal sperm analyses found no significant changes in the levels of FSH, LH, estradiol, sperm count, sperm motility, and sperm morphology. Previous studies on the action of circulating kisspeptin (a protein product of the Kiss1 gene) in the blood have reported a possible modulatory effect on menstruation, though with no observed correlation between circulating kisspeptin and reproductive hormones (Pinto *et al*., 2012). Similarly, our study found no significant correlation between the relative intensity of Kiss1 expression in the blood, reproductive hormones, and sperm parameters (Table 5).

Also, the testicular Kiss1 gene has been reported to play a role in sperm motility and transient sperm hyperactivity (Clavijo & Hsiao, 2018) and facilitate spermatogenesis by stimulating the production of androgen-binding receptors by Sertoli cells (Clavijo & Hsiao, 2018). This could explain the unchanged hormones and sperm parameters observed in our study as the expression of the testicular Kiss1 gene was not significantly altered. Our findings agree with studies from Idris *et al*. (2012), which also reported no significant changes to the levels of LH and testosterone following treatment with *HS.* However, our study disagrees with Nwabufo & Olusanya (2017), who observed significantly decreased LH and FSH levels, howbeit mild, following treatment with HS. The reason for this could be attributed to the study duration and the type of extract used.

## CONCLUSION

In summary, our study demonstrated that MEHS was able to alter the expression level of the Kiss1 gene in the blood but not in the testis. Also, MEHS did not cause any significant changes to hormone levels, oxidative profile, and sperm parameters. From our study, the detectable downregulated Kiss1 gene in the blood appeared to have no direct effect on the reproductive hormones and sperm parameters, indicating that the expression of Kiss1 in the blood may not be directly involved in the modulatory effect of kiss/Kiss1r in the hypothalamus-pituitary-gonadal axis. Furthermore, we hypothesize that the antioxidant constituents of HS (Ismail *et al*., 2008; Cisse *et al*., 2009; Okereke *et al*., 2015) may have played a role in protecting the testis and inhibiting the direct effect of Kiss1 gene modulation on the sperm parameters, tissue oxidant status and histopathology.

### Recommendation

Our study shows no evidence of any complementary effect on the reproductive hormones, testicular histology, sperm parameters and testicular antioxidative levels due to changes in Kiss1 gene expression in the blood. Further research needs to be carried out to understand the mechanism behind this effect and to isolate active phytochemicals from HS that may be responsible for altering the expression levels of the Kiss1 gene in the blood and modulating its role on the testes. More importantly, there is need for a chronic study with varying doses of HS to authenticate the claims of this present study.
